# Formulation and characterisation of a self‐nanoemulsifying drug delivery system of amphotericin B for the treatment of leishmaniasis

**DOI:** 10.1049/iet-nbt.2018.5281

**Published:** 2019-04-17

**Authors:** Momin Khan, Akhtar Nadhman, Walayat Shah, Imran Khan, Masoom Yasinzai

**Affiliations:** ^1^ Department of Biotechnology Quaid‐I‐Azam University Islamabad Pakistan; ^2^ Department of Pharmaceutical Technology Institute of Pharmacy Centre for Chemistry and Biomedicine (CCB) University of Innsbruck Innsbruck Austria; ^3^ Institute of Basic Medical Sciences, Khyber Medical University Peshawar Pakistan; ^4^ Institute of Integrative Biosciences, CECOS University of Science and Information Technology Peshawar Pakistan; ^5^ Division of Cancer Epidemiology and Management National Cancer Center‐809 Madu‐dong Ilsan‐gu, Goyang‐si Gyeonggi‐do 0‐769 Republic of Korea; ^6^ Centre for Interdisciplinary Research in Basic Sciences, International Islamic University Islamabad Islamabad Pakistan

**Keywords:** nanomedicine, drops, microorganisms, electrokinetic effects, cellular biophysics, drug delivery systems, monolayers, drugs, diseases, self‐nanoemulsifying drug delivery system, topical routes, oral routes, SNEDDS formulation, mucus permeation study, cell permeation, leishmaniasis treatment, amphotericin B, zeta potential, Caco‐2 cell monolayer, vortex, sonication, droplet size, Caco‐2 cell viability, antileishmanial activity, promastigotes, amastigotes, Leishmania parasites

## Abstract

This study was aimed to develop a self‐nanoemulsifying drug delivery system (SNEDDS) for amphotericin B (AmB) potential use in leishmaniasis through topical and oral routes. Two formulations, formulation A and formulation B (FA and FB) of AmB loaded SNEDDS were developed by mixing their excipients through vortex and sonication. The SNEDDS formulation FA and FB displayed a mean droplet size of 27.70 ± 0.5 and 30.17 ± 0.7 nm and zeta potential −11.4 ± 3.25 and −13.6 ± 2.75 mV, respectively. The mucus permeation study showed that formulation FA and FB diffused 1.45 and 1.37%, respectively in up to 8 mm of mucus. The cell permeation across Caco‐2 cells monolayer was 10 and 11%, respectively. Viability of Caco‐2 cells was 89% for FA and 86.9% for FB. The anti‐leishmanial activities of FA in terms of IC_50_ were 0.017 µg/ml against promastigotes and 0.025 µg/ml against amastigotes, while IC_50_ values of FB were 0.031 and 0.056 µg/ml, respectively. FA and FB killed macrophage harboured Leishmania parasites in a dose‐dependent manner and a concentration of 0.1 µg/ml killed 100% of the parasites. These formulations have the potential to provide a promising tool for AmB use through oral and topical routes in leishmaniasis therapy.

## 1 Introduction

Leishmaniasis is a neglected tropical disease [1], the clinical forms of which ranges from minor dermatological lesions to harsh disfiguring ulcers [cutaneous leishmaniasis (CL) and mucocutaneous leishmaniasis (MCL)] and fatal systemic infections in the spleen and liver (visceral leishmaniasis). Each form of the disease is caused by a different species of macrophage harboured protozoan parasite of genus Leishmania including *Leishmania tropica*, *Leishmania major*, *Leishmania amazonensis*, *Leishmania donovani*, *Leishmania mexicana and Leishmania infantum.* Leishmaniasis is a major health problem worldwide with a potential risk to 350 million people in 98 countries and approximately two million fresh cases are reported every year [2, 3].

Present challenges and threats in leishmaniasis chemotherapy include availability of limited drugs, emerging resistance to the drugs, targeted drug delivery to macrophages, toxicity and scarcity of cost‐effectiveness. The gold standard drug in leishmaniasis therapy is still an open question which changes from patients to patient, area to area and species to species of the parasite [4, 5]. Pentavalent antimony has long been the drug of choice and still plays a major role in leishmaniasis treatment. However, resistance to antimonials has been reported in some parts of the world [6]. Amphotericin B (AmB) has been effective in antimony resistant strains of Leishmania and is the drug of choice in antimonial resistant cases of leishmaniasis [7, 8, 9]. However, the use of AmB in leishmaniasis therapy is limited to its parental route only which has been frequently reported with dose‐dependent adverse side effects like thrombophlebitis, rigor, chills, myocarditis and nephrotoxicity [8, 10, 11]. Several formulations of AmB, e.g. lipid complex, colloidal form and liposomal form have been developed to reduce the adverse effects but their administration is still parental [9, 10]. In this scenario, strategies to increase therapeutic efficacy of AmB seem to be more promising as compared to the discovery and development of new drugs. This is why the organisation Drugs for Neglected Diseases initiative (DNDi) has chosen AmB to develop its topical dosage form for CL [12].

Drug‐delivery systems for leishmaniasis therapy are crucial as many active pharmaceutical modalities cause serious adverse effects when administered non‐specifically. Nanobiotechnology‐based drug delivery systems provide solution to this problem by delivering drugs specifically to the target infectious site. A few examples of such delivery system include liposomes, niosomes, nanodisks, nanoemulsions, solid lipid nanoparticles, polymeric nanoparticles and polymeric drug conjugates [2].

Poor bioavailability is a big challenge to prepare an oral dosage formulation of hydrophobic drugs [13]. Self‐emulsifying drug delivery systems (SEDDS) provides a platform to such drugs to enhance their bioavailability and reduce the toxicities by enabling the drug for oral and other non‐invasive routes. SEDDS are basically isotropic mixture of oil/lipids, surfactants, occasionally co‐solvent or co‐emulsifiers and the drug [14, 15]. The SEDDS formulations emulsify under conditions of mild agitation, similar to the condition being encountered in the gastrointestinal tract (GIT) [16]. These formulations can also emulsify on body tissues having some wet dispersion environment like vaginal, buccal, nasal and ocular mucosa. Several names have been given to SEDDS formulations on the basis of droplet size like self‐microemulsifying drug delivery system (droplet size 100–250 nm) and SNEDDS (self‐nanoemulsifying drug delivery systems: droplet size below 100 nm) [17]. The small size of the nanodroplets helps the drugs to pass efficiently through different barriers of the GIT. This property also helps the drugs to spread in effective way over the mucosal lining covering various anatomical sites [18, 19, 20]. Keeping in view the potential of SEDDS to solubilise hydrophobic drugs like AmB and imparting several beneficial pharmaceutical and pharmacokinetic profiles to the parent drug, this project was designed to develop AmB loaded SNEDDS formulations for oral and topical routes. The main objective was to increase the therapeutic efficacy of AmB against Leishmania.

## 2 Materials and methods

### 2.1 Materials

AmB was purchased from Sanova Pharma GesmbHA‐1110 Vienna. Cremophor RH40, Caprylic acid, Cremophor EL, were purchased from Sigma Aldrich, Vienna, Austria. Tween 80 and PEG 300 were purchased from Roth, Graz, Austria. Fungizone was purchased from Germany manufactured by Bristol‐Myers Squibb pharmaceuticals Ltd Swords, Country Dublin Ireland. Both Captex 300 as well as Captex 355 were a gift for research purpose from Abitec, USA. All other compounds were of analytical standard and obtained from commercial supplier sources from time to time.

### 2.2 Solubility studies

The solubility of AmB was determined by adding 1 mg of AmB in 1 ml of oils, surfactants and co‐surfactants in 2 ml eppendorf tubes. After vortex mixing the samples were kept in water bath at 37°C for 24 h. After that samples were centrifuged at 4000 rpm for 10 min to remove the un‐dissolved AmB. Then, 100 µl sample from each tube was taken in a microtiter plate and quantified at 382 and 405 nm wavelengths by using TECAN Infinite M200, Austria GmbH.

### 2.3 Preparation of blank and AmB loaded self‐nanoemulsifying formulations

For the preparation of SNEDDS, various oils, surfactants and co‐surfactants, listed in Table 1, were substantially homogenised by vortex mixer as reported by Kollner *et al.* with slight modification [21]. After sonication, the formulations were checked visually for turbidity and phase separation. The tendency of spontaneous emulsification and the progress of emulsion droplets were also visually observed by dissolving 1–2% (w/v) of emulsion in aqueous media. Afterwards, the most promising SNEDDS formulations were used for droplet (globule) size, zeta potential measurement analysis by dynamic light scattering with Zeta sizer (HSA 3000 Malvern Instruments Ltd, UK). Among these, the best formulations were selected to incorporate different concentrations of AmB (w/v) starting from 0.1 to 1%. The AmB loaded formulation were subjected to centrifugation at 13,000 rpm and characterised further for turbidity, phase separation and zeta potential over 24 h at 25 and 37°C. Afterwards, the best formulations were selected for further study keeping in view the pharmacokinetic and pharmacological properties of the excipients, size, polydispersity index (PDI) and stability of the formulations. The formulations are represented as FA and FB.

**Table 1:** Component and composition of excipients of SEDDS formulations in (%) values

Excipients	Captex 300	Cremophor EL	Cremophor RH40	Tween 80	DMSO	AmB
FA	20	45	—	—	35	0.4
FB	20	—	35	10	35	0.4

PDI: polydispersity index, DMSO: dimethyl sulfoxide, AmB: amphotericin B.

### 2.4 Mucus diffusion studies

The mucus diffusion ability of SNEDDS formulations was evaluated on porcine GIT mucus by rotating tube experimental method described earlier by Dünnhaupt *et al.* [22] and Pereira de Sousa *et al.* [23]. Briefly, silicon tubes (of 3 cm length and 3 mm diameter) were filled of mucus (100–120 mg). The mucus was filled up to 1 cm. Then, 100 µl of 0.05% lumogen red labelled SNEDDS formulation in phosphate buffer saline (PBS) (pH 6.8) was added and the tube was closed with rubber stopper. Silicon tubes of only mucus and 100 µl of PBS buffer was used as blank. The tubes were then rotated horizontally at 50 rpm at 37°C for 6 h and kept in −80°C freezer for overnight. Next day, the frozen tubes were cut into eight slices of ∼2 mm length and each was placed into a separate eppendorf tube. To trace the concentration of penetrated lumogen labelled SNEDDS, 200 µl dimethyl sulfoxide (DMSO) was poured to every tube containing the slice. The tubes along with samples were sonicated for 1 h in dark in order to dissolve the mucus and penetrated lumogen. Following sonication, the tubes were centrifuged at 13,000 rpm. Then 100 µl of supernatant of each samples was shifted to 96‐well microtiter plate for measurement of lumogen fluorescence intensity at *λ*_ex_  = 578 nm and *λ*_em_  = 613 nm using TECAN. The amount of lumogen permeated in each slice of silicon tube was calculated as percentage in comparison to the amount of lumogen red applied initially to the mucus.

### 2.5 In‐vitro transport of SNEDDS across the Caco‐2 cells monolayer

The study was conducted on the monolayer of Caco‐2 cells cultured onto the polyester plates (12‐well transwell plates). Then the cells were incubated in standard humidified condition of 5% CO_2_ at 37°C in minimal essential medium (MEM) with 20% foetal bovine serum (FBS). The growth of cells was monitored for a period of 21 days. The medium was changed after each 48 h. The integrity of cells monolayer was evaluated by measuring transepithelial electrical resistance (TEER) with EVOM instrument (Sarasota, FL). Cells monolayer having TEER values of about 400 Vcm^2^ were included in the study. Cells were washed with 500 µl PBS. Formulations (conc. 0.5% w/v) were prepared in Hanks balanced salt solution (HBSS) which served as transport medium. Afterwards, 500 µl of HBSS was added to the apical and 1.2 ml to the basolateral chambers of the culture plate. The medium of donor apical compartment was exchanged with 500 µl of 0.5% (m/v) of formulations after equilibration at 37°C for 1 h. Fungizone in the same concentration was used for comparison. Samples for analysis were regularly withdrawn from basolateral chambers and replaced by same amount of HBSS after every 30 min for a period of 4 h. The TEER values were again recorded at the end. The amount of AmB of both SNEDDS formulations and Fungizone transported via the cells monolayer was detected by HPLC. A Hitachi EliteLa Chrom HPLC system with L‐2130 pumps, L‐2200 autosampler and L‐2450 photodiode array and UV‐detector was used. A column C18 (250 × 4 mm, 5 µm) was utilised as stationary phase. The solvent system (40% methanol, 43% acetonitrile and 17% EDTA/water) was used at a flow rate of 1 ml per minute at room temperature.

### 2.6 Toxicity study on Caco‐2 cell lines

The in‐vitro resazurin viability assay was performed on Caco‐2 cell lines to assess the cytotoxicity of FA and FB, as reported by Jennings *et al.* [20] and O'Brien *et al.* [24]. Caco‐2 cells were plated in a 24‐well culture plate (density = 1 × 105 cells/well) in red MEM being supplemented with 10% (v/v) FBS and penicillin/streptomycin (100 units/0.1 mg/l) and later cultured for 14 days. One day before the assay, cells were washed three times with PBS. After that white MEM was added to the cells. The concentration of formulations was kept 0.5% in 500 μl in white MEM. White MEM served as a negative control while 1% (m/v) Triton X‐100 served as positive control. After 3 h incubation cells were again washed with PBS. After washing 5% (m/v) resazurin was added. The cells were later incubated for 2 h and fluorescence of the supernatant was measured at 540 nm excitation and 590 nm emission wavelengths by TECAN. The same procedure was repeated for 24 h toxicity studies. The following formula was used to calculate cells viability:

$$\mathrm{Cell}\;\mathrm{viablity}\;\left(\%\right)=\frac{\mathrm{Experiment}\;\mathrm{value}-\mathrm{negative}\;\mathrm{cotronl}}{\mathrm{Positive}\;\mathrm{control}-\mathrm{negative}\;\mathrm{control}}\times 100$$

### 2.7 Release study

UV–Vis spectrophotometer (Shimadzu 2600, Japan) was used to measure the drug release from SNEDDS without a membrane between release medium and oily droplets. Blank SNEDDS without the drug were prepared as a control. The auto‐zero corrections were made on the UV–Vis spectrophotometer in order to remove the background absorbance of the SNEDDS in release studies. Standard curve was generated by dissolving 0.2 mg AmB in 1 ml methanol and mixed overnight by a Thermomixer, at 2000 rpm at room temperature. Dilutions were made and measured on UV–Vis spectrophotometer at 405 nm.

The blank SNEDDS of formulation A was emulsified in 1 : 300 (v/v) ratios in water. The measurement was made in a quartz cuvette at 405 nm. After adjusting the auto‐zero for the emulsified blank SNEDDS, the measurement of the AmB loaded SNEDDS was performed. The drug‐loaded SNEDDS was emulsified in the same manner as discussed above. Readings were taken at time intervals of 1, 2, 3, 4, 5, 10, 15, 20, 30, 40, 50 and 60 min. All readings were taken in triplicate. Release of AmB from SNEDDS was evaluated [25].

### 2.8 Spreading studies over damaged skin and buccal mucosa

The spreading potential was investigated over damaged skin and buccal mucosa model of porcine. The buccal mucosa was removed carefully from buccal cavity and skin was taken from the ear and cheeks and immediately frozen at −20°C. The epidermis from skin was removed as reported by Walker *et al.* [26]. Fresh formulations were made with 1% fluorescein diacetate for spreading studies. A volume of 4 µl/cm^2^ of the SNEDDS was applied in the centre of the tissue. To make the fluorescence visible, UV lamp (excitation wavelength = 366 nm) was also placed. Pictures were taken after 5, 30 and 60 min to determine the spreading potential over the buccal mucosa and skin.

### 2.9 Anti‐leishmanial assays of the SNEDDS formulations

The anti‐leishmanial activities of the AmB loaded SNEDDS were assessed against the promastigote/amastigotes cultures of *Leishmania tropica*, (*L. tropica*). This was performed through in‐vitro analysis by using 2‐(4, 5‐dimethyl‐2‐thiazolyl)‐3, 5‐diphenyl‐bromide (MTT; Sigma, St. Louis, MO, US) viability colorimetric assay according to predefined protocol [27].

### 2.10 Anti‐leishmanial activity against intracellular amastigotes

For this experiment, human macrophages were isolated by the Ficoll–Gastrografin^®^ method as reported by Nadhman *et al.* [28, 29]. Briefly, macrophages were cultured in 24‐well chamber culture plates along with microscope slides to a density of 1 × 10^4^ cells/well. Cells were then incubated for adherence with chamber slides for 24 h. The monolayers were then infected with metacyclic *L. tropica* promastigotes at a ratio of 10 : 1 (parasite/macrophage) and were incubated at 37°C for seven days. Cells were then washed with PBS to remove the non‐phagocytosed parasites. Afterwards, the SNEDDS formulations FA and FB were added at a concentration of 0.01, 0.05 and 0.1 µg/ml each to a separate plate. After 24 h incubation chamber slides were fixed with methanol and stained with 4% Giemsa. The percentage of infected macrophages, as a minimum of 100 macrophages in each well was counted, and the percent inhibition was calculated.

### 2.11 Statistical analysis

The experiments were performed in triplicates. Statistical analysis was done by using SPSS 22 and confirmation with GraphPad Prism version 5. The data is shown as mean and standard deviation. To determine significant mean and interaction effect, *t* ‐test was performed for the comparison of the marginal means. *P*  < 0.05 was used to define significant results.

## 3 Results

### 3.1 Solubility studies of AmB

The solubility of AmB was determined in various solvents, oils, surfactant and co‐surfactants. AmB was found to have good solubility in the co‐solvent DMSO (data not shown). Thus, DMSO, Captex 300, Cremophor EL were selected as excipients for SNEDDS formulation FA and DMSO, Captex 300, Cremophor RH40 and Tween 80 were selected as excipients for SNEDDS formulation FB.

### 3.2 Preparation and characterisation of SNEDDS with and without AmB

Several SEDDS formulations were prepared and characterised for their emulsifying profile. The formulations were characterised for phase separation at different centrifugation rate, stability at different temperature ranges (4°C, room temperature and 50°C), size, PDI and zeta potential. The composition of most promising SNEDDS formulations FA and FB are summarised in Table 1 and other properties (visual appearance, phase separation, size, PDI and zeta potential) are given in Table 2.

**Table 2:** Characterisation of blank and AmB loaded SEDDS

Formulations	Size, nm	PDI	Zeta potential, mV	Phase separation at 13,000 rpm	Appearance
FA blank	25.66	0.108	−14.4 ± 4.47	no	white transparent
FA‐AmB	27.70	0.187	−11.4 ± 3.25	no	yellow transparent
FB blank	28.63	0.151	−12.3 ± 2.67	no	white transparent
FB‐AmB	30.17	0.171	−13.6 ± 2.75	no	yellow transparent

PDI: polydispersity index.

The oil (Captex 300), non‐ionic surfactants (Cremophor EL, Cremophor RH 40 and Tween 80) and the solvent (DMSO) showed best results for the preparation of pre‐concentrates of FA and FB. The SNEDDS formulation FA exhibited a mean droplet size of 27.70 ± 0.5 nm, PDI 0.187 and zeta potential −11.4 ± 3.25 mV while FB exhibited a mean droplet size of 30.17 ± 0.7 nm, PDI 0.171 and zeta potential of −13.6 ± 2.75 mV. The stability and phase separation of the formulations were also assessed time to time at different temperature for a period of one month. There was no significant change in the size and phase separation of both formulations at different temperature. The droplet/globule size of the SNEDDS formulation FA remained in the range of 24.24–29.30 nm with PDI range 0.112–0.241, while that of formulation FB remained in the range of 26.28–34.45 nm with PDI 0.142–0.253. Also, a slight change was observed in size and PDI when the dispersion medium was changed from water to PBS, HBSS and MEM there was slight change in size and PDI (data not shown).

### 3.3 Mucus permeation study

**Fig. 1:**
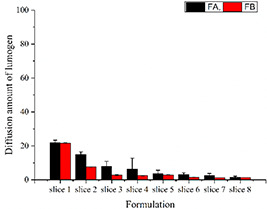
Diffusion of lumogen labelled SNEDD formulation (FA) and formulation (FB) in mucus (p < 0.05)

Lumogen red was incorporated into both SNEDDS formulation to determine their mucus diffusion potential. As shown in Fig. 1, diffusion through the first slice of FA was 22%. It decreased gradually and was 1.45% in last slice of silicon tube. The diffusion of FB was 21.6% in the first slice while it was 1.37% in the last slice. The net charge on mucus is negative while both of these formulations had negative zeta potentials (FA: −11.4 ± 3.25 mV and FB −13.6 ± 2.75 mV). The negative repulsive charges of both mucus and SNEDDS nano‐globule allowed the diffusion of lumogen labelled AmB loaded formulation up to the last slice of silicon tubes.

### 3.4 Permeation across Caco‐2 cells

**Fig. 2:**
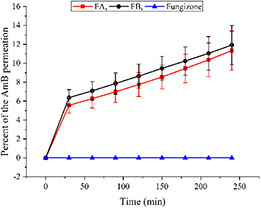
Permeation of AmB from SEDDS formulation FA, FB and Fungizone

The in‐vitro permeation studies of formulations FA and FB were compared with an equivalent amount of AmB in Fungizone (AmB‐sodium deoxicholate). Thus, 0.5% of SNEDDS preconcentrate was used. Caco‐2 cells monolayer efficiently transported 10% AmB from FA and 11% from FB to basolateral chamber after 4 h incubation. It is interesting to note that the transport of AmB was 0% from Fungizone for this time period (Fig. 2). The percentage of AmB transport from both formulations increased with the passage of time. In the first 1 h, it was faster as compared to the rest of time. From absorption point of view, this property was observed to be good as to prevent the loss of AmB from GIT.

The TEER values of cell monolayer were not changed after the experiment (data not shown). This means that the integrity of the cell monolayers was maintained during the entire time period of permeation study of AmB from SNEDDS formulations.

### 3.5 Toxicity study of formulations on Caco‐2 cell lines

**Fig. 3:**
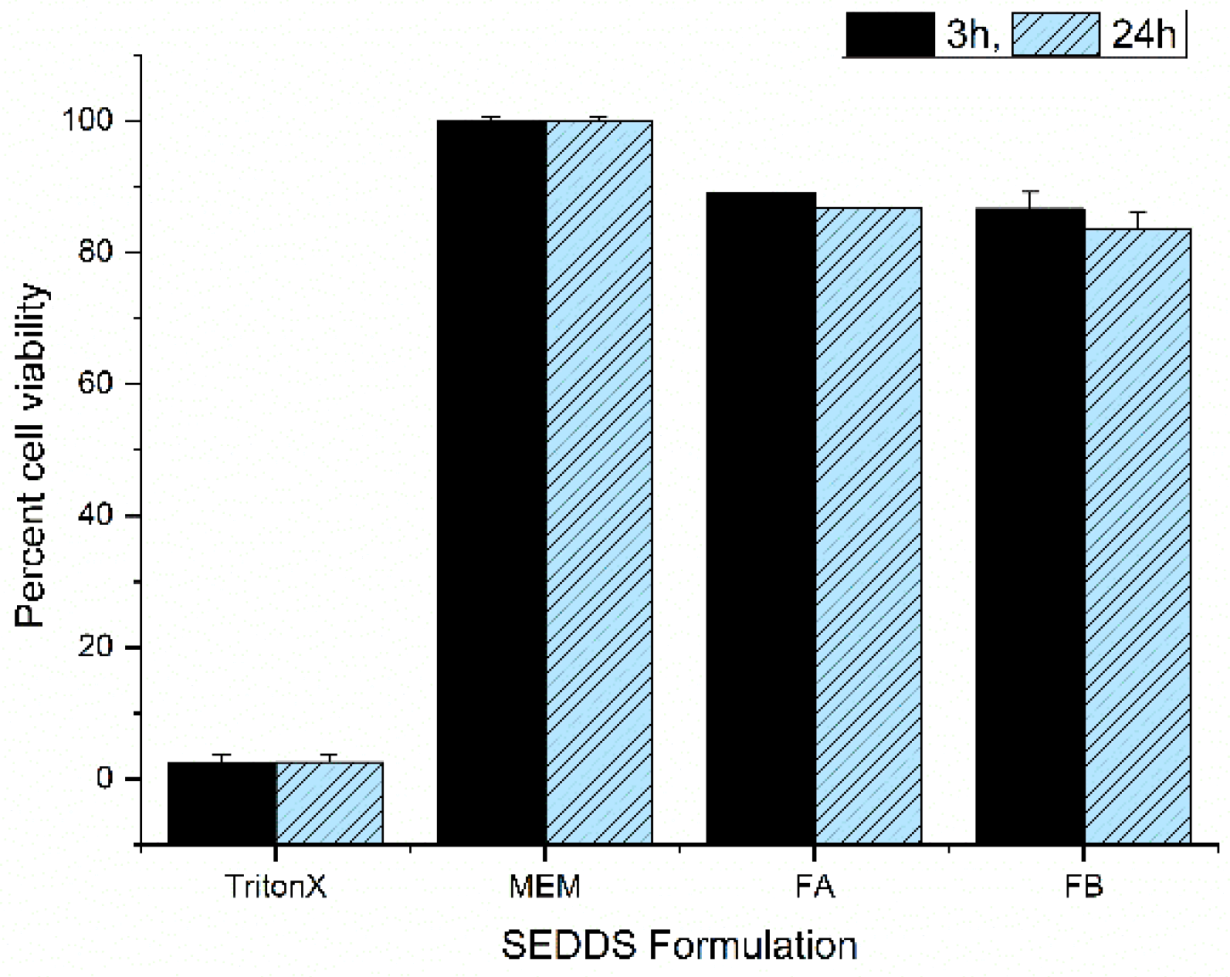
Viability of Caco‐2 cells using 0.5% of SEEDS formulations in comparison to triton X‐100 (p < 0.05)

Caco‐2 cells have the potential of differentiation into a monolayer acquiring the morphology and function of enterocytes (main cell type of small intestine), therefore this cell line was chosen to evaluate the toxic effect of SNEDDS on cells viability [30]. Cytotoxicity of the 0.5% AmB loaded SNEDDS formulations was evaluated. As shown in Fig. 3, the viability of Caco‐2 cells was 89% for FA and 86.94% for FB for 3 h study while it was 82% for FA and 80.79% for FB after 24 h study.

### 3.6 Release study of the AmB from SNEDDS

**Fig. 4:**
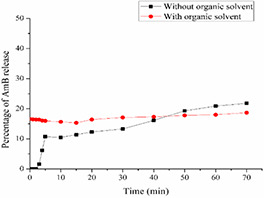
AmB release graph of SNEDDS formulated with and without organic solvent (DMSO)

The release study of only one formulation FA was assessed. For this purpose, two formulations of FA were developed, one with organic solvent (DMSO) and the other without organic solvent. The release of AmB began at almost 15% and went just 2% higher in the end. AmB loaded SNEDDS without organic solvent showed an increased release in comparison to SNEDDS with organic solvent (Fig. 4).

### 3.7 Spreading study of SNEDDS over buccal mucosa and damaged skin model

**Fig. 5:**
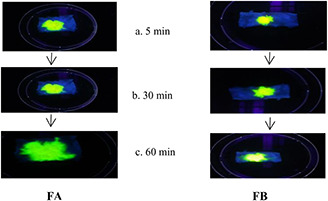
Spreading of SNEDDS formulations A and B (FA and FB) on damaged skin dermis. Images were taken at time interval ***(a)*** 5 min, ***(b)*** 30 min, ***(c)*** 60 min

**Fig. 6:**
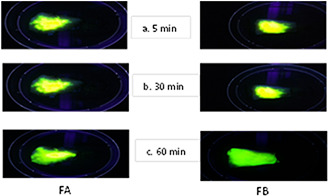
Spreading of SNEDDS formulations A and B (FA and FB) on buccal mucosa. Images were taken at time interval ***(a)*** 5 min, ***(b)*** 30 min, ***(c)*** 60 min

The spreading efficiency of SNEDDS formulations was assessed in order to explain its potential use in MCL and CL model of the disease. Ulcerated skin model was used, where the dermis was exposed to investigate the spreading behaviour of SNEDDS. As shown in Figs. 5 and 6, the spreading potential of FA and FB over buccal mucosa and damaged skin was remarkable.

FA and FB covered an area of 12 cm^2^ over buccal mucosa in 45 and 50 min, respectively, while these formulations covered an area of 14 cm^2^ over ulcerated skin model in 55 and 60 min, respectively. The spreading of the formulation over buccal mucosa is fast as compared to the damaged skin. These results demonstrate that the SNEDDS formulation of AmB can disperse and spread on damaged tissue providing the drug to adjacent ulcerated tissues.

### 3.8 Anti‐leishmanial assays of SNEDDS formulations

**Fig. 7:**
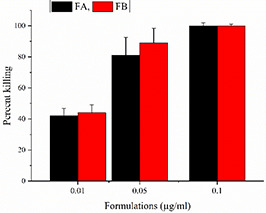
Percent killing of macrophage harboured Leishmania parasite in three different concentrations (p < 0.05)

Leishmania is a digenetic parasite. It passes from two morphological forms (promastigotes and amastigotes) during its life cycle. Therefore, the activities of SNEDDS formulation were assessed against both forms and additionally inside the macrophage infected stage of the parasite. The IC_50_ values of FA, FB and Fungizone are depicted in Table 3 and the percentage killing of parasite are given in Fig. 7. IC_50_ values of FA and FB (0.017 and 0.031 µg/ml) are much lower than Fungizone (0.61 µg/ml) against promastigotes and also for amastigotes. The efficacy of both the formulation was also exhibited in the macrophage harboured stage of the parasite. A dose‐dependent killing of the parasite was observed in this case. The percent killing of *L. tropica* for FA was 42, 81 and 100% at concentration of 0.01, 0.05 and 0.01 µg/ml, respectively. While the percent killing of *L. tropica* for FB was 44, 89 and 100% at concentration of 0.01, 0.05 and 0.1 µg/ml, respectively. These results clearly indicated that the SNEDDS formulation FA and FB effectively killed the parasites inside the macrophages.

**Table 3:** IC_50_ values of SNEDDS formulations FA and FB compared with Fungizone

*L. tropica* stage	Fungizone, µg/ml	FA, µg/ml	FB, µg/ml
promastigote	0.61 (±0.054)	0.017 (±0.005)	0.031 (±0.006)
amastigote	0.81 (±0.063)	0.025 (±0.003)	0.056 (±0.004)

*L. tropica* : *Leishmania* tropica, SNEDDS.

## 4 Discussion

The emerging resistance to antimonials has resulted in the increasing demand for use of second‐line drug AmB in leishmaniasis chemotherapy. However, the current use of AmB is solely parental in leishmaniasis.

The CL form of the disease somehow offers a spectrum of different routes ranging from parental to intra‐lesional. Parental route of AmB administration has been reported with various adverse reactions in patients. The WHO and other organisations such as DNDi recommend the topical treatment of CL and advise the parenteral route only if the topical therapy fails or cannot be performed. The idea behind this project was adopted from the concept of multiple applications of SEDDS and multiple clinical manifestations of leishmaniasis, for the development and characterisation of AmB loaded SEDDS. SEDDS provides an alternative tool for lipophilic drugs like AmB and offers the potential for enhancing drug absorption and oral bioavailability. Moreover, if provided with other suitable dispersion wet environment on other body sites (buccal, ocular, nasal and vaginal mucosa) SEDDS has the ability to adopt more convenient routes for its application [21, 31].

The pre‐concentrate of SNEDDS contain a blend of excipients in the form of oil or lipid, surfactant, co‐surfactant and, sometimes, co‐solvent. For drug delivery excipients the emphasis is on the use of those modalities which are safe for human consumption. Thus, we selected Captex 300, Cremophor EL, Cremophor RH 40, Tween 80 and DMSO for the pre‐concentrates of FA and FB. These excipients are listed in Generally Regarded as Safe category.

Captex 300 EP/NF (Glyceryl Tricaprylate/Tricaprate) is a medium chain ester and is generally recommended for its use in the manufacture of topical foams, creams, ointments and lotions as viscosity modifiers. This was selected for SNEDDS formulation FA and FB keeping in view its topical application in CL and MCL. Cremophor EL is synthetic non‐ionic surfactant. The main ingredient of this surfactant is polyethylene glycols ether. This is generally used to stabilise emulsions. In FA, we used it in 45% ratio together with 20% Captex and 35% DMSO. These ratios of excipients enabled AmB loading in hydrophobic core of droplets. Cremophor^®^ RH 40 is also a non‐ionic excipient. The main component of this surfactant is glyceryl polyethylene glycol oxystearate. Swab tests have demonstrated that Cremophor RH 40 is compatible with human skin mucus membranes [32]. It was used in FB in 35% ratio along with 20% Captex 300, 10% Tween 80 and 35% DMSO. This ratio loaded 0.4% AmB in the hydrophobic core of preconcentrate of FB. Tween 80 is a hydrophilic non‐ionic surfactant and emulsifier. The hydrophilic groups in this compound are polyethers. Tween 80 is a common excipient in various human dosage forms [33]. There are reports that Tween 80 is capable of enhancing the permeability of numerous drugs in vitro in Caco‐2 cells suggesting its potential role for its use in oral formulation [32]. In our study, Caco‐2 cells monolayer efficiently transported 10% AmB from FA and 11% from FB to basolateral chamber of the culture plate after 4 h incubation. The enhanced permeation of AmB in FB may be partly attributed to the presence of Tween 80.

The selection of DMSO in both formulations was due to two reasons. First, the solubility of AmB in DMSO, which dissolved maximum amount of AmB among all the solvents assessed for solubility study. Second, its potential to penetrate the skin and other membranes without harming them and could carry other excipients into biological systems. DMSO is mainly used in topical application of pharmaceuticals, as an analgesic, anti‐inflammatory and an antioxidant [19]. Keeping in view, the end use of the current formulations for topical applications in CL and MCL, DMSO was the best choice.

Excipients of the drug delivery should be non‐toxic systemically and to tissues of the body. In‐vitro evaluation of toxicity is important for erythrocytes and enterocytes [34]. Caco‐2 cells are used for intestinal permeability studies since the differentiated cells have the same morphological features as the human intestines. Both FA and FB were found non‐toxic to Caco‐2 cells. The viability of Caco‐2 cells was 89% for FA and 86.94% for FB for 3 h study. Thus, it is assumed that both of these formulations will be safe for oral route. The topical use of these formulations in CL will be safer because in such case they will not encounter the intestine and systemic barrier.

AmB has poor gastrointestinal absorption and negligible bioavailability when administered orally due to its hydrophobicity. Any SNEDDS formulation developed, for intended oral route usage should be assessed for mucus permeation and transport study across intestinal cell lines [34]. For this purpose, both the formulations were subjected to mucus permeation and transport studies across the Caco‐2 cell lines. The results show that FA and FB travelled up to the last slice of silicon tube (8 mm) filled with mucus. Mucus has negative charge while both of our formulations FA and FB displayed zeta potential as −11.4 ± 3.25 and −13.6 ± 2.75 mV, respectively. This permeation can be attributed to the fact of repulsion of same charges and net permeation to the last end of slice [35, 36]. Two factors seem to have played its role in the diffusion of formulation in the mucus, i.e. first small size of the droplet/globule and second, negative zeta potential of the droplets. Only diffusion in the mucus is not enough to get the desired goal of bioavailability. A best candidate nano‐formulation should be able to transport the drug across intestine. For this purpose, transport of formulations across Caco‐2 cell lines was carried out. We compared the transport of AmB from SNEDDS formulation and Fungizone (parental drug) and the results obtained showed improved properties. The transport of AmB across Caco‐2 cells was 10% from FA and 11% from FB after 4 h incubation, while it was 0% from Fungizone. Based on the results it can be suggested that AmB loaded SEDDS formulation via oral routes could be of potentially alternative strategy in near future. However, it may notice in this study that whether AmB alone was transported to basolateral chamber or it was transported in the globular form. A simple diffusion process releases the drugs from the SEDDS. The drug simply diffused out of the lipophilic phase into the aqueous phase. The log *P* of the drug, ionic interactions between the drug, SNEDDS components and the hydration process of the carrier system determined the release mechanism of the drug. When the drug molecules reached the surface of the oil droplets they have to pass the interfacial barrier in order to meet the aqueous medium [25].

Leishmaniasis manifests itself in three major clinical forms. Two of its form is cutaneous and mucocutaneous. The integrity of the skin or mucus membrane is damaged and the parasites harbour macrophages inside the dermis layer [2, 7]. The spreading efficiency of SNEDD formulations was assessed in order to explain its potential use in MCL and CL model of the disease. For this purpose, damaged skin model was used, where the dermis layer was exposed. Both the formulation exhibited remarkable spreading on both the models. Thus, one can utilise these formulations in MCL and open ulcerative lesion of CL. It is important to mention that CL manifests itself in many others forms including dry non‐ulcerated lesions. In such cases, the topical application of this SNEDDS formulation will not be appropriate. In such cases, the oral routes may offer more fruitful results than topical. However, a combination of both topical and oral routes may also be considered in case of complex CL. Before clinical trials of AmB loaded SNEDDS formulation, animal model study of CL is under consideration of our research group to find the conclusive evidence for such cases.

The therapeutic efficacy of a formulation can only be established if it achieves the desired goal. This goal for SNEDDS formulation of AmB, in leishmaniasis is the effective killing of the parasite in both of its morphological forms, i.e. promastigote and amastigote. In the current study, antileishmanial potential was evaluated for SNEDDS formulations against both forms and additionally inside the macrophage infected stage of the parasite. The IC_50_ values of SEDD formulations were far less than Fungizone. This means that SNEDDS formulations are more effective in fewer amounts compared to Fungizone and less quantities of the AmB will be required to kill the Leishmania parasite. This characteristic will reduce the toxicity‐related problems associated with high dosages of AmB. Furthermore, the SNEDDS formulations effectively released AmB in parasite‐infected macrophages and killed 100% Leishmania parasites at 0.1 µg/ml concentration. Thus, it is established that both the formulation can effectively get the desired goal of killing the parasites.

## 5 Conclusion

Current challenges in the classical leishmaniasis chemotherapy can be addressed by the application of SEDDS technology which provides a vehicle to poorly soluble drugs to enhance their bioavailability and reduce the toxicities by enabling the drug for oral and other non‐invasive topical routes. SNEDDS formulations FA and FB have the potential to provide a promising tool for AmB for its use through oral route in visceral leishmaniasis, CL and MCL. Also, both of these formulations can provide a non‐invasive topical route for the local administration of AmB in the treatment of CL and MCL.
